# Supplementary material for: A mechatronic shape display based on auxetic materials
Source: Nat Commun. 2021 Aug 6;12:4758. doi: 10.1038/s41467-021-24974-0 (PMC8346568; doi:10.1038/s41467-021-24974-0)
Supplement: Supplementary file 2 — Description of Additional Supplementary Files [file 41467_2021_24974_MOESM2_ESM.docx]

Supplementary Dataset 1: The three main parts required to make the base of the display. Suitable for laser cutting.

Supplementary Dataset 2: The parts to make simple mould of the right shape to hold the shape surface for casting in silicon

Supplementary Dataset 3: The parts used to make the socket that holds the end of the linear actuator to the bottom of the surface.

Supplementary Dataset 4: The pattern that we water cut to make the surface of the display.

Supplementary Dataset 5: Corresponds to the participant responses of experiment 1.

Supplementary Dataset 6: Corresponds to the participant responses of experiment 2.

Supplementary Dataset 7: Corresponds to the participant responses of experiment 3.

Supplementary Movie 1: Supplemental movie showing how the auxetic shape display is constructed and operated.
